# Amino Acid Deprivation in Glioblastoma: The Role in Survival and the Tumour Microenvironment—A Narrative Review

**DOI:** 10.3390/biomedicines12112481

**Published:** 2024-10-29

**Authors:** Keven Du, Leila Grocott, Giulio Anichini, Kevin O’Neill, Nelofer Syed

**Affiliations:** 1 Imperial College School of Medicine, Imperial College London, London SW7 2AZ, UK; keven.du20@imperial.ac.uk (K.D.); leila.grocott17@imperial.ac.uk (L.G.); 2 Department of Brain Sciences, Imperial College London, London SW7 2AZ, UK; kevin.oneill@imperial.ac.uk (K.O.); n.syed@imperial.ac.uk (N.S.)

**Keywords:** glioblastoma, amino acid deprivation, high-grade gliomas, cancer metabolism

## Abstract

Background: Glioblastoma is the most common and aggressive primary brain tumour, characterised by its invasive nature and complex metabolic profile. Emerging research highlights the role of amino acids (AAs) in glioblastoma metabolism, influencing tumour growth and the surrounding microenvironment. Methods: This narrative review synthesises recent pre-clinical studies focusing on the metabolic functions of AAs in glioblastoma. Key areas include the effects of AA deprivation on tumour growth, adaptive mechanisms, and the tumour microenvironment. Results: The effects related to arginine, glutamine, methionine, and cysteine deprivation have been more extensively reported. Arginine deprivation in arginine-auxotrophic glioblastomas induces apoptosis and affects cell adhesion, while glutamine deprivation disrupts metabolic pathways and enhances autophagy. Methionine and cysteine deprivation impact lipid metabolism and ferroptosis. Tumour adaptive mechanisms present challenges, and potential compensatory responses have been identified. The response of the microenvironment to AA deprivation, including immune modulation, is critical to determining therapeutic outcomes. Conclusions: Targeting AA metabolism offers a promising approach for glioblastoma treatment, with potential targeted drugs showing clinical promise. However, the complexity of tumour adaptive mechanisms and their impact on the microenvironment necessitates further research to optimise combination therapies and improve therapeutic efficacy.

## 1. Introduction

### 1.1. Glioblastoma: The Therapeutic Landscape

Glioblastoma (GB), the most common primary brain tumour, is a highly aggressive cancer due to its invasiveness and complex molecular and metabolic profiles [1]. The standard of care involves surgical resection and post-operative chemoradiotherapy [2,3]. The goal of surgery is to achieve a Gross Total Resection (GTR) in every case where this is possible [3], and there is evidence that radiotherapy increases survival and delays neurological deterioration, offering some degree of control over disease progression [4]. Chemotherapy in GBs consists mainly of alkylating agents that can cross the blood–brain barrier [5,6]. The so-called “Stupp protocol”, combining radiotherapy with a regimen of Temozolomide (TMZ), has become the standard of care in most countries [3,6], with other alkylating agents being second- or third-line treatment choices. However, its disease control or survival advantage is unsatisfactory [7,8]. Even for cases receiving full treatment, the average survival rate is 15 months, and no significant prognostic improvement has been achieved in the past twenty years [3].

Alternatives under investigation include targeted therapies, such as bevacizumab, an anti-angiogenic agent that inhibits vascular endothelial growth factor (VEGF) and has shown promise in slowing tumour progression [9]. Immunotherapies, including checkpoint inhibitors like nivolumab, aim to enhance the immune system’s ability to recognise and destroy tumour cells [10]. Additionally, chimeric antigen receptor (CAR) T-cell therapy, which reprograms T-cells to target specific tumour antigens, is being explored in clinical trials [11]. Finally, gene therapy targets particular mutations that affect both oncogenetic and metabolic pathways in GBs, although the challenge of finding the appropriate delivery strategy persists [12,13].

### 1.2. Oncometabolites and Glioblastoma

Metabolic changes in glioblastomas, like those in other types of cancer, play a pivotal role in reprogramming the cell to adapt to a new environment and altered growth rate [14]. In recent years, metabolic targeting strategies have offered promising alternatives to conventional treatments [14,15]. The emerging research on cancer metabolism has highlighted the metabolism’s pivotal role in cell sustainability, proliferation, growth, and microenvironment [14,15]. Therapeutic approaches include targeting glucose metabolism, the tricarboxylic acid (TCA) cycle, and growth factors. Glucose metabolism is a hallmark of glioblastoma’s reliance on glycolysis (the “Warburg effect” [16]) and can be significantly shifted by blocking hexokinase 2 (HK2) [17] or glucose transporter 1 (GLUT1) [18]. Targeting key enzymes within the TCA cycle, such as isocitrate dehydrogenase (IDH), has shown the potential to disrupt tumour cell metabolism [19,20]. Inhibiting oxidative phosphorylation through agents like metformin [21,22] and targeting the mammalian target of the rapamycin (mTOR) cascade [23,24], which regulates cell growth and metabolism, are approaches being studied as promising therapeutic avenues linked to metabolic pathways. Although not exempt from criticisms regarding the practicality of its clinical deployment [25], this metabolic approach supposedly weakens the tumour’s energy production and biosynthetic capacity.

Some of the critical mutations related to glioblastomas and the relative changes in concentration of oncometabolites are reported in Table 1.

While a large amount of research has been focused on glucose metabolism, one promising avenue has focused on depriving glioblastoma cells of amino acids (AAs), which otherwise engage in compensatory mechanisms supporting glioblastoma metabolism despite nutrient deprivation [32]. The present narrative review focuses on the role of amino acids in glioblastomas’ catabolic and anabolic processes and their effect on the surrounding microenvironment.

## 2. Metabolism of Amino Acids in Glioblastoma and the Effects of Specific Amino Acid Deprivation

The role of metabolism in sustaining tumour proliferation and invasion has been explored in gliomas since the 1970s [33,34]. Glucose metabolism and the related tricarboxylic acid (TCA) cycle have been extensively investigated due to glucose’s primary role in sustaining the glioma metabolic pathways. Glycolysis and oxidative phosphorylation are linked to several complex metabolic processes involving lipids, AAs, transcription factors, epigenetic modifications, and signalling pathways, all of which are altered in gliomas and contribute to tumour proliferation and invasion [19].

Several AAs have been investigated in the context of glioma metabolism, adaptability, and growth. However, the research was focused on specific para-physiological phenomena, such as altered metabolite concentrations, ligand-dependent transporters, and receptors [35,36,37,38]. More recent years have witnessed a renewed interest in the key role of AAs in the metabolic profile of gliomas, their compensatory role in the context of nutrient deprivation or metabolic stress, and their potential as molecular markers. The published literature is overwhelmingly pre-clinical, and most authors have focused on studying one specific amino acid. Targeting one AA is a common choice, and it is often directly related to the mutations and metabolomic profile of the examined glioblastoma cell line. Studying AA deprivation in cell cultures serves more than one purpose: on one side, it helps define potential dietary targets to improve the response to cancer treatment, and on the other, it helps with discovering pathways activated by the cancer cells as a compensatory mechanism under stress conditions, therefore identifying novel pathogenetic and potentially therapeutic targets.

The most relevant studies from the past five years are discussed below, but this is an expanding research domain, and the list is intended to be partial. It is plausible that more findings will soon emerge from the international literature.

### 2.1. Arginine

Arginine is a semi-essential AA. Most mammals convert it into citrulline while releasing nitric oxide (NO) [36]. Arginine has been extensively studied in different types of cancer, and it seems to be linked to several oncogenic pathways, including the mammalian Target of Rapamycin (mTOR) cascade [37]. Astrocytes predominantly express inducible nitric oxide synthase (iNOS), which converts arginine to l-citrulline and NO and is activated in response to inflammation. The arginase 1 (ARG1) enzyme supports the conversion of arginine to ornithine and urea, the former being a crucial component for the synthesis of polyamines [38].

Arginosuccinate synthetase 1 (ASS1) and arginine-succinate lyase (ASL) are crucial enzymes for arginine biosynthesis from citrulline and aspartate. A few groups have highlighted the potential for arginine deprivation treatments targeting arginine-auxotrophic glioblastomas, which are ASS1-deficient and, therefore, unable to synthesise arginine intracellularly [39,40,41]. These glioblastomas are vulnerable to arginine-depleting agents such as pegylated arginine deiminase (ADI-PEG20). Glioblastomas, which are non-arginine-auxotrophic and proficient in ASS1 production, may also be affected by ADI-PEG20, particularly in combination with ionising radiation [38].

Arginine deprivation is also thought to influence tumour cell viability and invasiveness, ultimately affecting cell growth rather than causing cell death, resulting in reversible changes following the cessation of therapy [39]. However, some have recently explored evidence to create combination therapies involving arginine deprivation to ensure prolonged, irreversible effects of treatment both in vivo [38] and in vitro [39,41].

One mechanism of anti-tumoral cell death occurs through apoptosis and structural changes to actin filaments of glioblastoma cells. Some authors showed that co-treatment with arginine deprivation and canavanine incubation enhances this process [39]. Canavanine is an antimetabolite, non-proteinogenic AA sharing a similar structure to arginine, though differing through the replacement of carbon with oxygen in the methylene group. Co-treatment with canavanine incubation in an arginine-deprived medium appears to result in the accumulation of cleaved caspase 3 in both U87MG (ASS1-proficient) and U251MG (ASS1-deficient) human GB cell lines [42,43]. A subsequent reduction in B-cell leukaemia/lymphoma 2 protein (Bcl-2) was noted in the U251MG cells. Blc-2 is a protein commonly cleaved by caspase 3 and known for its role in apoptosis inhibition. This finding highlights the role of a caspase-dependent apoptotic pathway in ASS1-deficient cells [39]. This apoptotic pathway affected glioblastoma (GB) cells but not healthy ones. Pre-treating arginine-deprived cells with canavanine prevented tumour growth once arginine was reintroduced, whereas arginine deprivation alone demonstrated reversibility. Moreover, the integrity of actin filaments and cell adherence likely plays a role in the pathway of proliferation inhibition. For example, a notable finding was the reduction in actin filaments such as lamellipodia in both U87MG and U251MG cell lines. Further changes to actin structure include the destabilisation of the actin cytoskeleton and the appearance of actin-containing aggregates, indicative of cell death [39]. Given that these changes were only seen in therapy with an arginine-free medium, arginine deprivation is the suspected responsible underlying mechanism. This hypothesis is also supported by the pre-incubation with canavanine, which prevents reversibility of cell proliferation [39].

Another mechanism of action inhibiting pro-tumoral proliferation in the context of canavanine co-treatment involves reducing cell adhesion to the extracellular matrix. Cell adhesion assays identified a 75% reduction in cell adhesion following combination therapy. However, immunocytochemical staining and immunoblotting did not reveal any changes to the levels of proteins involved in cell adhesion, such as vinculin and talin, suggesting a defect in cell adhesion originating from a source other than protein signalling pathways [39].

Besides apoptosis and changes to actin structure, arginine deprivation appears to cause mitochondrial dysfunction and dysregulated DNA repair in glioblastoma cells. Authors have examined the effects of combined treatment with arginine deprivation therapy and cyclin-dependent kinase (CDK) inhibitors in ASS1-deficient glioblastoma cells due to the CDK activity in a variety of cancers [41]. Notably, transmission electron microscopy confirmed the presence of mitochondrial damage and autophagy of mitochondria. This damage was reflected by the subsequent reduction in oxygen consumption rates in arginine-auxotrophic glioblastoma cell lines [41]. Furthermore, marked structural changes within mitochondria, such as vacuolisation originating from the endoplasmic reticulum (ER), resulting in vacuoles containing debris of organelle remains, affect tumour metabolism by altering signalling pathways. For example, an increase is seen in integral ER proteins such as calnexin and glucose-regulated protein 78 (GRP78) following CDK inhibition and canavanine co-treatment, respectively, as well as protein-coding genes such as the Activating Transcription Factor 4 (ATF4), all of which reflect ER stress [39,41]. These proteins are responsible for regulating protein folding, thereby producing significant stress to the ER from the accumulation of misfolded proteins, leading to reduced phosphorylation and, in turn, reduced activation of Adenosine MonoPhosphate (AMP)-dependent kinase (AMPK), inhibiting its role in energy homeostasis [39]. Another effect on a signalling pathway is the increased phosphorylation of Stress Activated Protein Kinase/c-Jun N-terminal kinases (SAPK/JNK) and Mitogen-Activated Protein Kinase (MAPK) p38, which increases pro-apoptotic signalling pathways. Moreover, arginine deprivation, alongside CDK inhibition, results in increased beta-galactosidase levels, a marker of cellular senescence. DNA damage, a common cause of cellular senescence, is reflected through increased Growth Arrest and DNA Damage-inducible 45 (GADD45) protein. Therefore, arginine deprivation may affect DNA damage, creating mitochondrial dysfunction and subsequent impairment of tumour metabolism and, ultimately, cell death [41].

### 2.2. Glutamine and Glutamate

Glutamine is a non-essential amino acid involved in the tricarboxylic acid cycle. It is involved in the biosynthesis of nucleotides, glutathione (GSH), and other non-essential AA [44,45]. Under normal physiological conditions, glutamine enters the cell through a dedicated transporter and can be converted to glutamate by the enzyme glutaminase. Glutamate can subsequently be converted into α-ketoglutarate and has been shown to supply the tricarboxylic acid cycle under glucose-deprived conditions [45]. Glutamate is also exchanged with cystine in the extracellular compartment through a dedicated anti-porter, xCT, a physiological mechanism that also seems to play a crucial role in providing cystine for the synthesis of glutathione [46]. In cancer metabolism, glutamine seems to be the key nutrient the pathological cell becomes “addicted” to in the absence of glucose [47]. Glucose metabolism seems closely intertwined with AA metabolism, a characteristic highlighted in several other cancer subtypes [48]. The combined deprivation of glucose and glutamine causes a marked reduction in proliferation and leads to apoptosis and autophagy in glioblastoma stem-like cells [49]. Glutamine-deprived glioma cells showed decreased proliferative activity [50], an effect replicated when liver-derived glutaminase, which is not expressed in glioma cells, is transfected in glioma cell lines [51]. However, this effect is presumably counterbalanced by the tumour increasing glutamine synthase expression [52] and promoting autophagy through several strategies, including activation of Lyn kinase [53] and phosphoglycerate kinase (PGK1) [54]. Glutamine deprivation also causes increasing micropinocytosis, a possible compensatory process activated to scavenge for nutrients [55].

As mentioned, the glutamine pathway is activated in response to glucose deprivation and, when disrupted, it is presumed to cause a marked reduction in cell proliferation. Uncoincidentally, glutamine deprivation seems to affect the synthesis and action of several genes involved in glucose metabolism such as ECE1, EDN1, glPDHA1, PDHB, DLAT, and DLD genes, which is why a few studies also compared it with glucose deprivation [56,57].

Glutamine deprivation is linked to the expression of the Endoplasmic Reticulum to Nucleus Signalling 1/Inositol Requiring Enzyme 1 (ERN1/IRE1) in the context of glioblastoma [56], a gene coding for a protein that acts as a detector for unfolded proteins in the endoplasmic reticulum. Said detection activates a stress response, resulting in both unfolded proteins’ degradation and several stress-related genes’ activation [58]. In the context of ERN1 knocked-down glioblastoma cell lines, the proliferation is markedly reduced in an environment without glutamine and glucose, an aspect that seems to be directly correlated with a change in expression of ERN1-dependent genes, such as endothelin-1 (EDN1, downregulated), its receptors A and B (EDNRA, downregulated, and EDNRB, upregulated), and the endothelin converting enzyme (ECE1, upregulated, although more markedly in control cells than in ERN1 knocked-down cells) [56]. The endothelin protein and its related receptors are pro-mitotic and anti-apoptotic factors, specifically in glioblastoma [59]. ERN1 was also found to be directly linked to changes in the regulation of the genes belonging to the homeobox complex, which are involved in central nervous system embryology and are known to have a role in the proliferation of glioma cells both in paediatric [60] and adult [61] patients. While glutamine deprivation seems to promote a variable degree of upregulation or downregulation on several homeobox genes, ERN1 knocked-down cells showed more marked sensitivity and downregulation of the LIM homeobox 1 (LHX1) in response to glutamine deprivation [62]. However, while glutamine deprivation was found to downregulate the expression of genes related to glucose metabolism, including some of the subunits of the pyruvate dehydrogenase complex, ERN1 knocked-down cells altered some of these responses [57]. Overall, all these studies point towards a key role of ERN1 in glioma cell proliferation, a role which seems to affect ERN1-dependent genes to a different extent under glutamine deprivation.

Several other genes involved in glucose metabolism and Isocytrate DeHydrogenase 1 (IDH1)-mutant glial cell proliferation have been reported to respond to glutamine deprivation, including the N-myc Downstream Regulated Gene 2 (NDRG2), a member of the N-myc downregulation pathway acting as a tumour suppressor in the absence of glutamine [63]. Moreover, the glutaminase inhibitor JHU-083, a pro-drug that can penetrate the blood–brain barrier and is converted into 6-diazo-5-oxy-L-norleucin, caused a marked reduction in cell proliferation and downregulation of the mTOR pathway in animal models of IDH1-mutant gliomas [50]. This finding is particularly promising for clinical translational research, and JHU-083 is currently being evaluated for its first clinical trial.

Interestingly, the growth arrest-specific 6 (Gas6)–Axl signalling pathway, a known cascade where the tyrosine kinase Axl deactivates the proliferation promoter ligand Gas6 protein [64], is suspected of counteracting glutamine deprivation and promoting cell division even under nutrient-deprived conditions [65].

As mentioned above, converting glutamine to glutamate is one of the mechanisms decreasing cell survival and proliferation [51]. Glutamate is also exchanged with cystine to guarantee the synthesis of glutathione. The interdependency between glutamate, cysteine, and glutamine in glutathione synthesis is linked to telomerase reverse transcriptase (TERT). This enzyme is crucial for glioblastoma proliferation and is linked to the overexpression of glutamate–cystine ligase (GCLC) by way of increasing the expression of the Forkhead box protein O1 (FOXO1) transcription factor [66]. Its inhibition, mediated by deploying buthionine sulfoximine (BSO), seems to depress the proliferative activity. However, the activation of the MYC pathway partially compensates for this effect and prevents cell death. The adjunct of 6-diazo-5-oxy-L-norleucin, the active form of the pro-drug JHU-083, seems to have a synergistic effect on glioblastoma cell cultures and cause apoptosis [66]. Some authors have expressed the glutamine/glutamate interdependency in ratio form (Gln/Glu) and examined cell cultures with different profiles to test the response to AA deprivation [67]. When cells are deprived of glutamine, the expression of the glutamate/cystine transporter xCT is upregulated, as is that of Zinc finger E-box-binding homeobox 1 (ZEB1), a key transcription factor involved in cell invasion and stemness. When xCT is knocked down, the Gln/Glu ratio is reduced, the expression of ZEB1 is downregulated, and the proliferation and clonogenicity of the cell cultures are markedly reduced [67]. These findings not only highlight the importance of glutamine deprivation in reducing the proliferative activity of glioblastomas, but also suggest that the Gln/Glu ratio and the related expression of xCT/ZEB1 are potential molecular markers for more aggressive subtypes of glioblastoma. A recent study investigated glutamine metabolism’s heterogeneity in GB [68]. Using gene expression data, the authors identified 13 glutamine metabolism-related genes, such as MYC, Nuclear factor erythroid 2-related factor 2 (NRF2), and Phosphoinositide 3-Kinase (PI3K), and stratified GBs into two groups showing different overall survival and progression-free survival. More importantly, the combined used of glutaminase inhibitor CB839 and DiHydroArtemisinin (DHA), an anti-malaria agent with anti-cancerous activity based on increased oxidative stress, induced apoptosis and impaired the migration and proliferation of GB cells [68]. The role of CB839 was also previously explored by different authors, who showed the accumulation of intermediate metabolites in both the purine nucleotide pathway and a decreased level of TCA intermediates [69].

**Figure 1:**
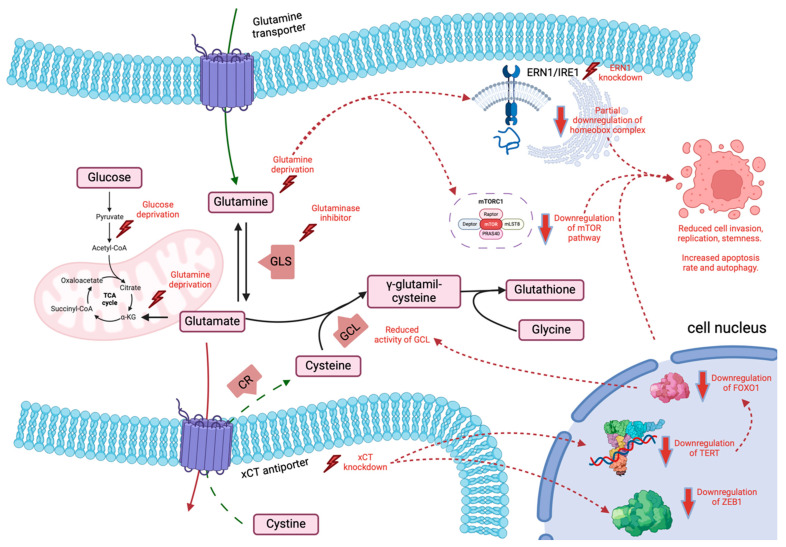
Schematics of the physiological role of glutamine and some pathways which are suspected to be affected by its deprivation in glioblastoma (see text for more details). GLS—Glutaminase; CR—Cystine Reductase; GCL—Glutamine–Cysteine Ligase. Picture created with BioRender.com.

In summary, glutamine seems to be a pivotal AA in the altered metabolism of GB, and its pathways are closely linked to those of glutamate, cysteine, and the TCA cycle, as reported by several authors and recently summarised in a review [70]. Glutamine deprivation inhibits cell metabolism and replication. However, several molecular cascades mediate this effect, and a few compensatory mechanisms must be considered when considering the prospect of glutamine deprivation for therapeutic strategies. Figure 1 depicts some of the described metabolic effects.

### 2.3. Methionine and Cysteine

Methionine and cysteine are often reported or considered together due to their involvement in a common biochemical pathway. Specifically, methionine is an essential amino acid that is converted into s-adenosyl-methionine (SAM) and subsequently into s-adenosyl-cysteine (SAH) through two enzymes, methionine adenosyl-transferase 2A (MAT2A) and a methyltransferase (MT), respectively. SAM is also an intermediate of the methionine recycling pathway which is, in turn, involved in the synthesis of polyamines and purines. Finally, cysteine is also a precursor of glutathione [71,72]

A known mechanism of cell death in vivo is related to ferroptosis, an iron-mediated lipid peroxidation process prevented by glutathione. The role of cystine (the disulfide form of cysteine) deprivation in accelerating ferroptosis and generalised lipid peroxidase-related cell death has been highlighted by several authors, both in vitro [73,74] and in vivo [75]. More recently, the simultaneous deprivation of methionine and cysteine was also found to reduce glutathione production drastically [72]. The key factor involved in the process, as demonstrated for other cancer cell lines [76,77], was found to be Ras-Sensitive Ligand 3 (RSL3), an inhibitor of the glutathione peroxidase 4 gene (GPX4). Cysteine and methionine deprivation also causes a change in the lipid and metabolic profiles within the tumour while sparing the surrounding brain tissue. A marked overall increased survival of the treated animals compared to the control group is also observed, which is a promising finding for potential therapeutic targets [72].

Besides glutathione and polyamine synthesis, methionine plays an important role in DNA methylation [78]. From a strictly metabolic perspective, methionine restriction seems to cause increased lipolysis, insulin sensitivity, reduced oxidative stress, and inflammatory response in different types of cancer [78].

Most authors observed that methionine restriction limits cell proliferation through several epigenetic, metabolic, and signalling pathways [79,80].

For example, methionine deprivation seems to cause a marked effect on DNA methylation in glioblastoma cell cultures [80]. Glioma-initiating cells cultured on methionine-deprived medium showed a generalised DNA demethylation with specific areas of hypermethylation affecting genes involved in signalling pathways for pluripotent stem cells. The corresponding silencing of several pluripotent stem cell markers was observed through microRNA, cDNA, and PCR analysis. Regarding the metabolic effect, methionine deprivation caused the upregulation of a series of genes involved in the physiological methionine pathway, specifically MAT2A and 2B, with a concomitant decrease in the concentration of the intermediate products, such as SAM. At the same time, cholesterol biosynthesis was reported to be reduced, and its excretion increased. This effect was not linked to hydroxy-methyl-glutaryl-CoA synthase 1 gene (HMGCS1) activity, as its expression was unaffected, and no change in methylation was observed. However, the sterol regulatory element-binding factor 2 gene (SREBF2), which targets a series of genes related to cholesterol metabolism (including HMGCS1), showed a hypermethylated profile. Moreover, the forkhead box protein M1 gene (FOXM1), an essential gene involved in fatty acid production, was also found to be downregulated [80].

### 2.4. Other Amino Acids

While several groups have studied the deprivation of some specific AAs, others have yet to be examined so extensively. As mentioned, the choice to study one AA is related to its involvement in molecular pathways specific to glioblastoma metabolism and to one cell line. This aspect again highlights the need for phenotype-specific and possibly even individually tailored therapies. Some of the effects of other AAs studied in the context of glioblastoma metabolism are reported here, but the list is by no means comprehensive.

Serine and glycine are semi-essential AAs reported to play a crucial role in one-carbon metabolism, specifically in cancer [81]. Serine can be synthesised from the glycolysis byproduct 3-phosphoglycerate (3-PG) through a series of passages and then converted to glycine by the enzyme serine hydroxymethyl transferase 1 (SHMT1) in the cytoplasm or 2 (SHMT2) in the mitochondria. When depriving glioblastoma cell lines of both serine and glycine, the glycolysis enzymes are overexpressed to stimulate the de novo synthesis of both AAs, a process mediated by the activation of the AMP-activated protein kinase (AMPK) and its action on the hypoxia-inducible factor 1-α (HIF-1α) [82]. The resulting hyperexpression guarantees increased tumour survival. The inhibition of the AMPK–HIF-1α pathway caused decreased serine levels, depressed glycolysis, and increased apoptosis in GB cell lines. High AMPK and/or HIF-1α levels in human GB specimens also correlated with worse prognosis [82].

Tryptophan is one of the most extensively studied AAs in cancer and neurodegeneration [83], which is why it has also been investigated in the context of glial tumours [84,85]. It seems to affect the tumour microenvironment, although it might impact the immune system in a more pro-tumoral direction (see below).

Some authors have studied the effect of multiple AA deprivation on cancer cell metabolism. For example, exposing U87MG and SF767 glioblastoma cell cultures to different combinations of amino acid depletion, specifically glutamine, arginine, and lysine in one group and methionine and cysteine in another group [86], caused changes in the expression of specific genes; however, these changes were more pronounced for some AAs than others. The study highlighted a more marked sensitivity to methionine and cysteine starvation, with consequent cyclooxygenase-2 (COX-2) induction, crucial for prostaglandin synthesis, promoting proliferation and angiogenesis. The process was found to be mediated through the p38 MAPK pathway and the transcription factor Sp1; both factors are known to increase proliferation in cancer [87]. Moreover, the COX-2 induction was directly linked to increased vascular endothelial growth factor (VEGF) expression, which promotes angiogenesis [86].

## 3. The Effect of Amino Acid Deprivation on the Tumour Microenvironment and Immunomodulation

Although interest is rapidly growing, the effect of AA deprivation on the tumour microenvironment is still poorly understood. Some of the aforementioned AAs tend to reduce tumour proliferation and survival, a process that is not only mediated by changes in the metabolic and oncogenic pathways within the cell, but also by altering the tumour microenvironment. However, deprivation of a specific AA can lead to various effects, some of which counteract the benefits.

For example, the metabolism of arginine is suspected to modulate immunosuppression. Glioma-Associated Macrophages/Microglia (GAMMs) metabolise arginine through both iNOS and ARG1 pathways, the former of which supports a pro-inflammatory, anti-tumoral environment and the latter bolsters an anti-inflammatory, pro-tumoral environment [38]. GAMMs often produce both iNOS and ARG1 but tend to predominantly produce one or the other [38,88]. GAMMs predominantly expressing iNOS encourage cytotoxic activity and production of NO and reactive oxygen species (ROS) [36,38]. Those GAMMs predominantly expressing ARG1 initiate angiogenesis, invasion, proliferation, and immunosuppression [36,38]. As mentioned above, ADI-PEG20 treatment is possibly linked to increased recruitment of GAMMs to the tumour site, with the strongest effects being observed in combination with ionising radiation (IR) [38]. Increased intracellular lipid-like body accumulation was observed in ADI-PEG20 monotherapy and combination therapy, which suggests an increase in phagocytic activity. ADI-PEG20 alone reduced the production of ARG1; however, when combined with IR, there was an additional effect of increased iNOS expression. Two microglial/macrophage pro-tumour gene clusters were identified, and mesenchymal and neural GB subtypes seemed to predominate cluster 1. Finally, ADI-PEG20 has been shown to increase the susceptibility of GB cells to IR via increases in NO production and stimulating GAMMs to switch from pro-tumoral to anti-tumoral activity [38].

**Figure 2:**
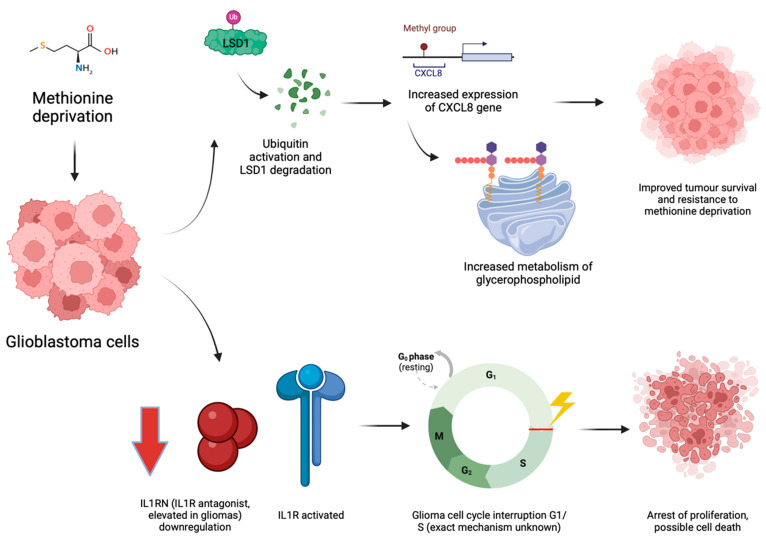
Conflicting effects of methionine deprivation in glioblastoma (see text for more details). The reduced expression of IL1RN, an antagonist of the IL1 receptor, causes cell cycle arrest and reduced proliferation. However, methionine deprivation also causes increased expression of CXCL8, which theoretically should attract neutrophils but also increases glycerophospholipid metabolism, giving a survival advantage to the cancer cells under stress conditions. Picture created with BioRender.com.

Similarly, methionine deprivation seems to affect specific interleukins’ expression or action, although the microenvironmental effect is unclear. For example, cells deprived of methionine seem to suppress the expression of Interleukin 1 Receptor Antagonist (IL1RN), one of the main factors known to be upregulated in gliomas and suspected to promote the progression of the cell cycle in tumour cells [79]. The exact mechanism linking methionine with reduced IL1RN expression and its biological effect on the tumour is unclear. Still, a cell cycle phase arrest at G1/S was observed in a methionine-deprived animal model after harvesting and analysing the tumour tissue using flow cytometry [79]. However, methionine deprivation could also be linked to a pro-tumoral and anti-immune effect, as it has been found to activate CXC interleukin 8 (CXCL8) pathways. CXCL8 is a chemokine promoting tumour proliferation, invasion, and migration in several types of cancers [89]. In the model proposed by Chang and colleagues, methionine starvation causes activation of the CBL–LSD1–CXCL8 pathway. Specifically, when glioma cells experience methionine starvation, the E3 ubiquitin ligase CBL (named after Casitas B-lineage Lymphoma) targets the Lysine-Specific histone Demethylase 1 (LSD1) for degradation via the ubiquitin–proteasome pathway. The resulting demethylation activity of LSD1 is therefore reduced, leading to increased mono-methylation at histone H3K4 sites on the CXCL8 gene, enhancing its expression. The elevated levels of CXCL8 are linked to an increased glycerophospholipid metabolism, altering membrane dynamics and signalling pathways crucial for cell survival, resulting in a poorer prognosis [90] (see Figure 2).

Similar conflicting results were observed on the local immune profile when different AAs were tested. For example, depletion of tryptophan was linked to T-cell death and is suspected to be an anti-immune compensatory mechanism activated by the tumour. Specifically, the overexpression of the IMPACT gene was increased in several cancer types, including glioblastoma, and this increased expression constituted a marked survival advantage under tryptophan deprivation [85]. IMPACT is a protein enabling ribosome binding activity and is directly related to the general control non-depressible 2 (GCN2) protein kinase. This protein kinase works as a sensor of AA depletion. The described altered gene profile causes decreased activity of the stress response pathways and increased activity in pathways involved in translational regulation, such as the mTOR pathway [85]. Rashidi et al. explored a tryptophan-deprived animal model of glioblastoma with a specific focus on the CD8 + T-cells [84]. The expression of GCN2 was knocked down in animal models of glioblastoma, causing a pronounced depletion of CD8 + T-cells. This effect was even more pronounced under tryptophan deprivation, and it is likely due to the reduced activity of protein kinase C-theta (PKCθ), which is critical for T-cell activation and survival. Overall, these results suggest that tryptophan deprivation, while having an anti-tumoral effect on the glioblastoma cells themselves, also impacts the survival of CD8 + T-cells, leading to decreased activation, increased cell death, and consequent local immunodeficiency [84]. All these effects have also confirmed that tryptophan depletion-related T-cell death is counterproductive and might have to be considered when exploring novel therapies against glioblastomas.

## 4. Conclusions and Future Perspectives

The present review highlights the intricate role of AAs in glioblastoma metabolism, emphasising how specific AAs like arginine, glutamine, methionine, and cysteine contribute to tumour growth, proliferation, and survival. The current literature evidence suggests that targeting AA metabolism, mainly through deprivation strategies, can reduce glioblastoma viability and impact the surrounding microenvironment. Arginine deprivation in arginine-auxotrophic glioblastomas triggers apoptotic pathways and affects cell adhesion, while glutamine deprivation disrupts key metabolic pathways and enhances autophagy in glioblastoma stem cells. However, the tumour’s adaptive mechanisms and potential compensatory responses, such as increased ER stress and altered gene expression, present significant challenges.

Future research should focus on understanding these compensatory pathways in greater detail and exploring combination therapies that could inhibit these survival mechanisms. The interplay between AA metabolism and the tumour microenvironment warrants further investigation, as modulating AA availability may alter immune responses and influence therapeutic outcomes. Ultimately, this expanding field offers promising avenues for developing more effective and targeted treatments for glioblastoma and a more stratified classification of tumours based on their molecular and metabolic profiles.

The most significant limitation of the current body of evidence is that all the studies are pre-clinical. Promising drugs for potential clinical trials include depleting agents to enhance deprivation [38], transporter inhibitors to reduce the supply of AAs to the altered metabolic pathways [67], or glutaminase inhibitors to depress key metabolic steps [68].

## Figures and Tables

**Table 1:** Summary of some of the most common mutations found in gliomas, with their effect on concentrations of relevant oncometabolites.

Mutation	Metabolic Effect	ONCOMETABOLITES		Effects on Tumour Proliferation
		Increased Concentration	Reduced Concentration	
IDH [26,27,28]	Altered TCA cycle	2-Hydroxyglutarate (2-HG)	Alanine, glutamine, glutamate	Inhibition of DNA and RNA demethylases; increased histone, DNA, and RNA methylation, leading to altered gene expression
1p/19q codeletion [29] *	N/A	N/A	Glutamate	Associated with IDH mutations; associated to improved response to therapy
EGFR [27,28]	Increased glycolysis	Lactate	N/A	Increased cell proliferation
PTEN [26]	Increased glycolysis	Lactate	N/A	Enhanced PI3K/AKT signalling, driving proliferation
mTOR [28]	Increased glycolysis	Acetyl-CoA, lactate	N/A	Upregulated biosynthesis (e.g., lipid, nucleotide synthesis) required for rapid tumour cell proliferation
Several mutations, including HIF-1α [30]	Increased fatty acid accumulation and metabolism	Fatty acids	N/A	Associated with increased tumour proliferation and resistance to alkylating agents
TERT [31]	Increased fatty acid accumulation and metabolism	Fatty acids	N/A	Associated with increased tumour proliferation and resistance to alkylating agents

IDH—Isocitrate Dehydrogenase; TCA—Tricarboxylic Acid; 2-HG—2-Hydroxyglutarate; EGFR—Epidermal Growth Factor Receptor; PTEN—Phosphatase and Tensin Homolog; mTOR—Mechanistic Target of Rapamycin; HIF-1α—Hypoxia-Inducible Factor-1 alpha; TERT—Telomerase Reverse Transcriptase. * Found in oligodendrogliomas.
